# ShinyOmics: collaborative exploration of omics-data

**DOI:** 10.1186/s12859-020-3360-x

**Published:** 2020-01-17

**Authors:** Defne Surujon, Tim van Opijnen

**Affiliations:** Biology Department, Boston College, Chestnut Hill, MA 02467 USA

**Keywords:** Systems biology, Visualization, Transcriptomics, Functional genomics, Data integration

## Abstract

**Background:**

Omics-profiling is a collection of increasingly prominent approaches that result in large-scale biological datasets, for instance capturing an organism’s behavior and response in an environment. It can be daunting to manually analyze and interpret such large datasets without some programming experience. Additionally, with increasing amounts of data; management, storage and sharing challenges arise.

**Results:**

Here, we present ShinyOmics, a web-based application that allows rapid collaborative exploration of omics-data. By using Tn-Seq, RNA-Seq, microarray and proteomics datasets from two human pathogens, we exemplify several conclusions that can be drawn from a rich dataset. We identify a protease and several chaperone proteins upregulated under aminoglycoside stress, show that antibiotics with the same mechanism of action trigger similar transcriptomic responses, point out the dissimilarity in different omics-profiles, and overlay the transcriptional response on a metabolic network.

**Conclusions:**

ShinyOmics is easy to set up and customize, and can utilize user supplied metadata. It offers several visualization and comparison options that are designed to assist in novel hypothesis generation, as well as data management, online sharing and exploration. Moreover, ShinyOmics can be used as an interactive supplement accompanying research articles or presentations.

## Background

Omics-profiling is becoming increasingly prevalent in many subfields in biology. For example, genome-wide transcriptomics have been used in studies of gene expression during embryonic stem cell differentiation, host-pathogen interactions, identification of biomarkers associated with antibiotic resistance and cancer disease progression [1–8]. Similarly, proteomic screens can identify proteins relevant for virulence, or cancer biomarkers [9–12]. Furthermore, phenotypic profiling using transposon insertion sequencing (Tn-Seq) in human pathogens has identified genes involved in colonization, infection, and intrinsic antibiotic resistance; and has been used in genetic interaction mapping [13–18].

Since genome-wide multi-omic profiling is paving the way to such varied and clinically relevant applications, considerable effort has gone into establishing analysis pipelines that process the resulting data. Tools such as DESeq2 [19] and MAGenTA [20] are used for statistical analysis of differential gene expression and fitness changes respectively. However, the volume of the analyzed data can make interpretation and comprehensive evaluation non-trivial. Moreover, these tools often do not accommodate easy incorporation of metadata pertaining to genes and/or experimental conditions. This makes it time consuming and labor intensive to apply custom analysis protocols on each dataset, especially if the user has limited programming experience.

Existing tools for user-friendly data exploration and visualization include Stemformatics [21], Metascape [22], and mixOmics [23]. Stemformatics is an online portal that assembles gene expression data from stem cell datasets. While it provides an interactive visual interface, Stemformatics is tailored for stem cell research, and hosts a specific and focused dataset that does not expand to fields other than stem cell research. Metascape does allow users to supply their own datasets (often in the form of a gene list extracted from differential expression or other omics profiling data), and can merge information from public databases as well as perform functional enrichment and network analyses. The heavy dependence on well-curated annotation and information on public databases can be a limitation for researchers working with less well-characterized organisms, where these annotations may not be readily available; or available to the user but not yet made public. Moreover, even though the user can provide gene lists extracted from different omics screens, these analyses are performed independently. mixOmics is an R package that allows the user to interact with and analyze their own (potentially unpublished) data with less reliance on public databases, and consider multi-omics data simultaneously. It provides multiple pipelines focused on dimensionality reduction and feature selection, which can be extremely valuable in determining what signatures are associated with for instance disease outcome. However, if a researcher’s interests are more specific, e.g. asking what expression changes are observed for a specific set of genes, a more customizable platform may be better suited.

To complement existing tools, we present ShinyOmics, a browser-based interface that allows for customizable visualizations of genome-wide profiling data, incorporating user-supplied metadata from genes and experimental conditions, and network connectedness of genes. It is straightforward to swap out the existing datasets loaded in ShinyOmics with user-generated custom data; e.g. standard output from DESeq2 can directly be incorporated. This feature of ShinyOmics also facilitates data management and sharing; for example, a lab can host a fully interactive instance of ShinyOmics with their own data making it accessible to collaborators across the world through a URL. This creates a convenient alternative over transferring and describing a large number of spreadsheets and data files between labs. Moreover, ShinyOmics can be deployed with new data obtained in a research project, as an interactive supplement that can be included in a manuscript submission, or academic presentation.

## Implementation

ShinyOmics was developed in R version 3.4.3 [24], using RStudio version 1.1.419 [25]. Running the app locally requires the packages ggplot2 [26] (v3.1.0), visNetwork [27] (v2.0.5), RColorBrewer [28] (v.1.1), igraph [29] (v1.2.2), heatmaply [30] (v.0.16.0), shinyHeatmaply [31] (v.0.1.0) and shiny [32] (v1.2.0).

An example of the app with data from [33–35] is available at [36]. The source code for the app and detailed usage notes can be accessed from [37]. Detailed usage notes are also provided in the aforementioned link.

There are three types of custom data that can be added; genome-wide profiling data, strain metadata, and network data. The main reference file for the app is “exptsheet.csv” under the “data” subdirectory. Any added experiment needs to be recorded in this file, with the corresponding profiling and metadata file locations specified. At minimum exptsheet.csv should have columns “Experiment”, “Time”, “Name”, “DataFile”, “Strain”, and “MetadataFile”. There can be as many additional columns as desired to record metadata of the experiments. For profiling data files, the standard output of DESeq2 can be directly transferred to the “data” directory. Alternatively, a file with at least the columns “Gene”, “Value” (e.g. log2 fold change of expression), and “padj” can be provided. While the data source can be any organism or strain, eukaryotic datasets with tens of thousands of genes are likely to cause significant lag in the application loading. We therefore recommend, in the case of eukaryotic data, filtering the dataset (based on the number and quality of reads, or variability among replicates) and working with only a subset of a few thousand genes at most. There needs to be one metadata file per strain, and the minimum requirement for each metadata file is one column labeled “Gene”. Each metadata file can have as many columns as desired, all selectors on the app will adjust accordingly. Finally, the networks should be specified as edge tables, with two columns: “source” and “target”, and be named “[Name]_Edges.csv” in the “data/networks/” subdirectory. The network statistics will be computed automatically.

When the app is first loaded in the browser, all data/metadata files and the experiment sheet will be screened and validated for the requirements mentioned above. If the files provided do not fit these specifications, pop up error messages will indicate what caused the validation to fail, in which file(s), and the app will load with no data.

## Results

We provide a version of ShinyOmics pre-loaded with multi-omic data from two human pathogens; *Streptococcus pneumoniae* and *Mycobacterium tuberculosis*. The *S. pneumoniae* dataset includes Tn-Seq and RNA-Seq data from two strains (TIGR4 and 19F) that were exposed to 1x Minimum Inhibitory Concentration (MIC) of kanamycin (KAN), levofloxacin (LVX), rifampicin (RIF), vancomycin (VNC) and penicillin (PEN) for 2–4 h [33]. Differential expression (DE) on the RNA-Seq data was evaluated as the fold change in transcript abundance comparing antibiotic conditions to a no-antibiotic control using DESeq2 [19]. Fitness change (dW) on the Tn-Seq data was evaluated comparing antibiotic to no-antibiotic conditions as described in [17]. The *M. tuberculosis* dataset includes microarray data [34] and proteomics data [35] under hypoxic conditions over a span of up to 20 days of culture in vitro. In its current configuration there are four panels that allow for different types of visualization: Single Experiment, Comparison of 2 Experiments, Comparison of All Experiments, and Network Visualization.

**Fig. 1:**
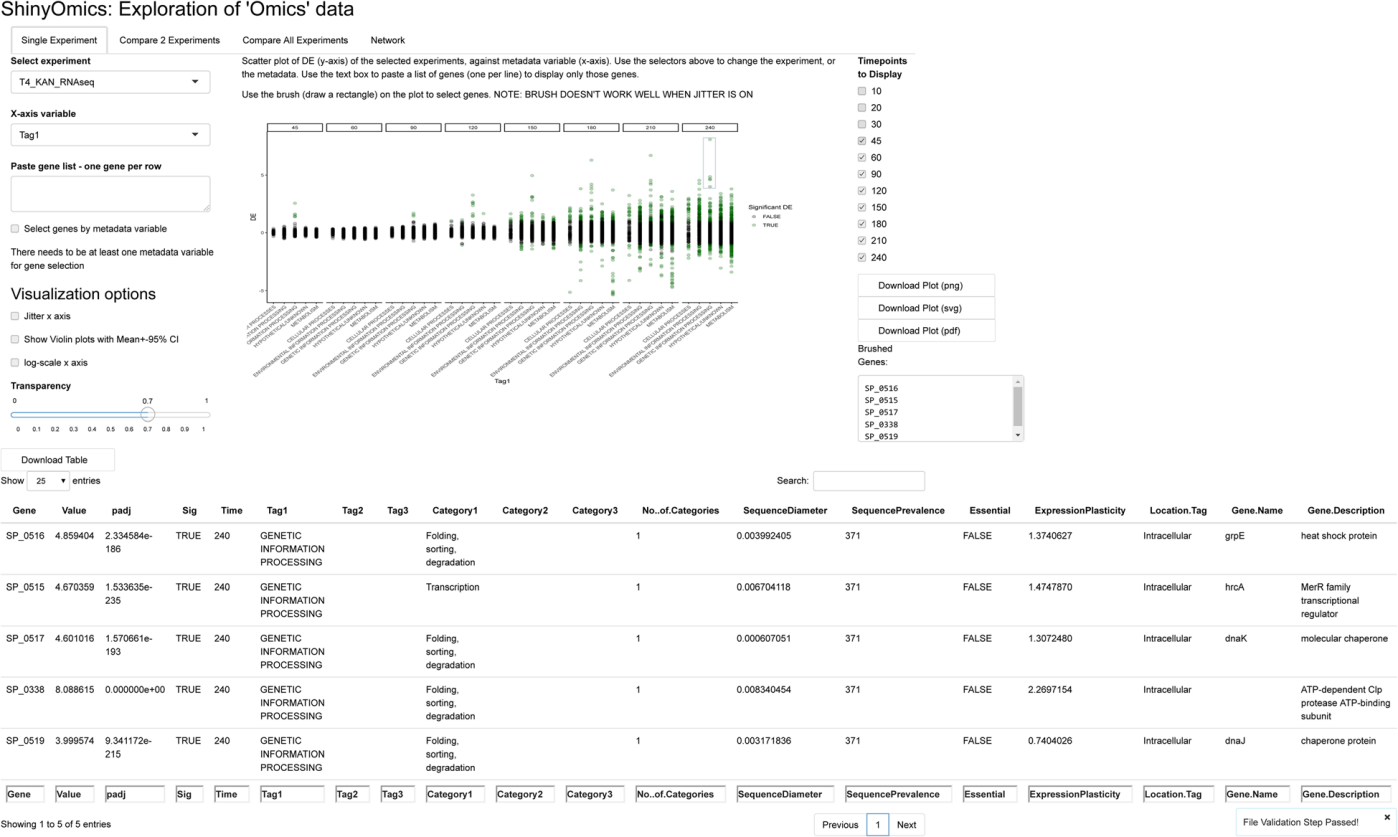
Single Experiment panel of ShinyOmics. The tabs above allow the user to navigate to different panels. On the left, there is an experiment selector (where options are populated from the experiment sheet supplied by the user), a gene list selector (when empty, all genes are displayed), a variable selector, and several visualization customization options. Here, the T4 kanamycin (“T4_KAN”) experiment is displayed as a scatterplot. Setting the x-axis variable to “Tag1” splits genes by functional Tag. 4 genes are brushed at timepoint 240 (blue rectangle), whose identity and metadata are displayed in the table (bottom)

In ShinyOmics the first panel is designed to explore relationships between a value associated with all genes (e.g. DE, dW, protein abundance) and any other user supplied metadata (Fig. 1). The metadata variables and their descriptions can be found in Additional file 1: Table S1. The user can include other genome-wide profile data (e.g. change in fitness, dW) in the metadata fields, or as a separate experimental data file. In the Single Experiment panel, DE is plotted against the selected metadata type. For instance, in the preloaded dataset, one can answer whether there are significant DE changes appearing in a specific cellular function, by selecting “Tag1” (primary functional tag of the gene) from the dropdown menu labelled “Variable” (Fig. 1). The resulting scatter plot has each gene as a point, with the categorical variable “Tag1” on the x-axis and DE on the y-axis. The plot is faceted by timepoints, i.e. each timepoint in the selected experiment is a separate panel. The user can select which timepoints to display or hide using the checkboxes on the right. There are several visualization tuning options, such as changing the transparency of points, or in the case of categorical x-axis variables, adding some noise (or “jitter”) to the x-coordinate of each point (such that individual points do not overlap) and/or superimposing a violin plot. It is also possible to display only a subset of genes by pasting a gene list in the text box (“Paste gene list”), subsetting the genes by a metadata variable (“Select genes by metadata variable”), or to select genes directly from the plot by dragging a rectangle to define a region of interest (or “brushing”) the plot. The brushed genes will be displayed in the table below. Clicking anywhere on the plot will reset the brushing. In the example provided, it is possible to identify a set of genetic information processing genes that are upregulated drastically when *S. pneumoniae* is exposed to kanamycin (Fig. 1). Kanamycin, an aminoglycoside, is a protein synthesis inhibitor that triggers the incorporation of erroneous amino acids during protein synthesis, leading to an accumulation of misfolded proteins [38]. In *S. pneumoniae* TIGR4, the Clp protease ATP-binding subunit (SP_0338) is upregulated 256-fold (Fig. 1), indicating a response by this organism to alleviate the antibiotic stress through the destruction of misfolded proteins. This is accompanied by the simultaneous upregulation of chaperones dnaK and grpE (SP_0517 and SP_0516), whose function it is to repair denatured and misfolded proteins [39].

**Fig. 2:**
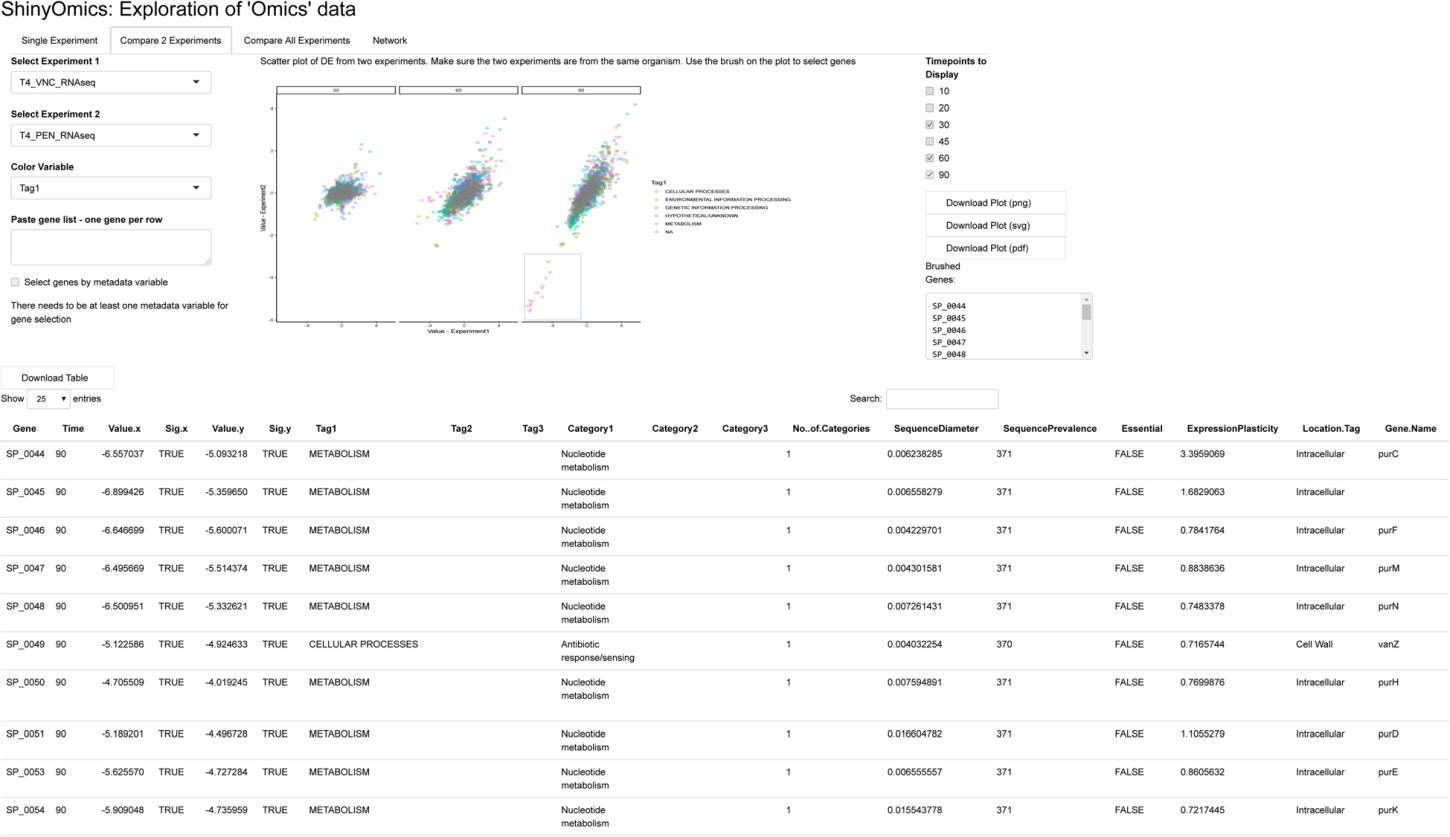
Comparison of 2 experiments. On the left are selectors for the two experiments to be compared, and a color variable. Here, DE from vancomycin (VNC) and the penicillin (PEN) are being compared for T4. Blue box on the plot indicates a set of brushed points. The table below the plot (cropped) displays all available information regarding the brushed points

The Compare 2 Experiments panel allows for quick pairwise comparisons of experiments (Fig. 2). Here, one can plot the DE of one experiment against another, for the timepoints that are in common in both experiments. There is a selector for the color of the points (e.g. one can color each gene by functional category, or any other metadata feature). The plot is brushable, similar to the Single Experiment panel. As an example, the DE of two antibiotics are compared in Fig. 2. Vancomycin and penicillin are both cell wall synthesis inhibitors, and the transcriptomic changes in response to these antibiotics appear highly correlated, especially in the later timepoints (Fig. 2). This global similarity in transcriptional profiles is unique to the PEN-VNC pair, and is not observed when comparing antibiotics of different classes. In contrast, at 90-min a group of genes are brushed (SP_0044-SP_0054, Fig. 2) belonging to the category “Nucleotide metabolism” that turn out to be downregulated across most of the tested antibiotics, including the RNA synthesis inhibitor Rifampicin, and the DNA synthesis inhibitor Levofloxacin. This set of genes are part of the purine biosynthesis pathway, and their downregulation might point to a common antibiotic response in *S. pneumoniae* TIGR4.

It is also possible to see whether different systems under the same condition harbor similar responses using the Compare 2 Experiments panel. Comparison of Tn-Seq and RNA-Seq data from *S. pneumoniae* antibiotic experiments and a comparison of microarray and proteomic data from *M. tuberculosis* shows a lack of similarity in the responses in the different screens (Additional file 1: Figure S1). This is in accordance with previous findings that systems-level data are often quite distinct, and different systems should not be taken as substitutes of one another, but rather complementary parts of the organism as a whole [18, 40].

**Fig. 3:**
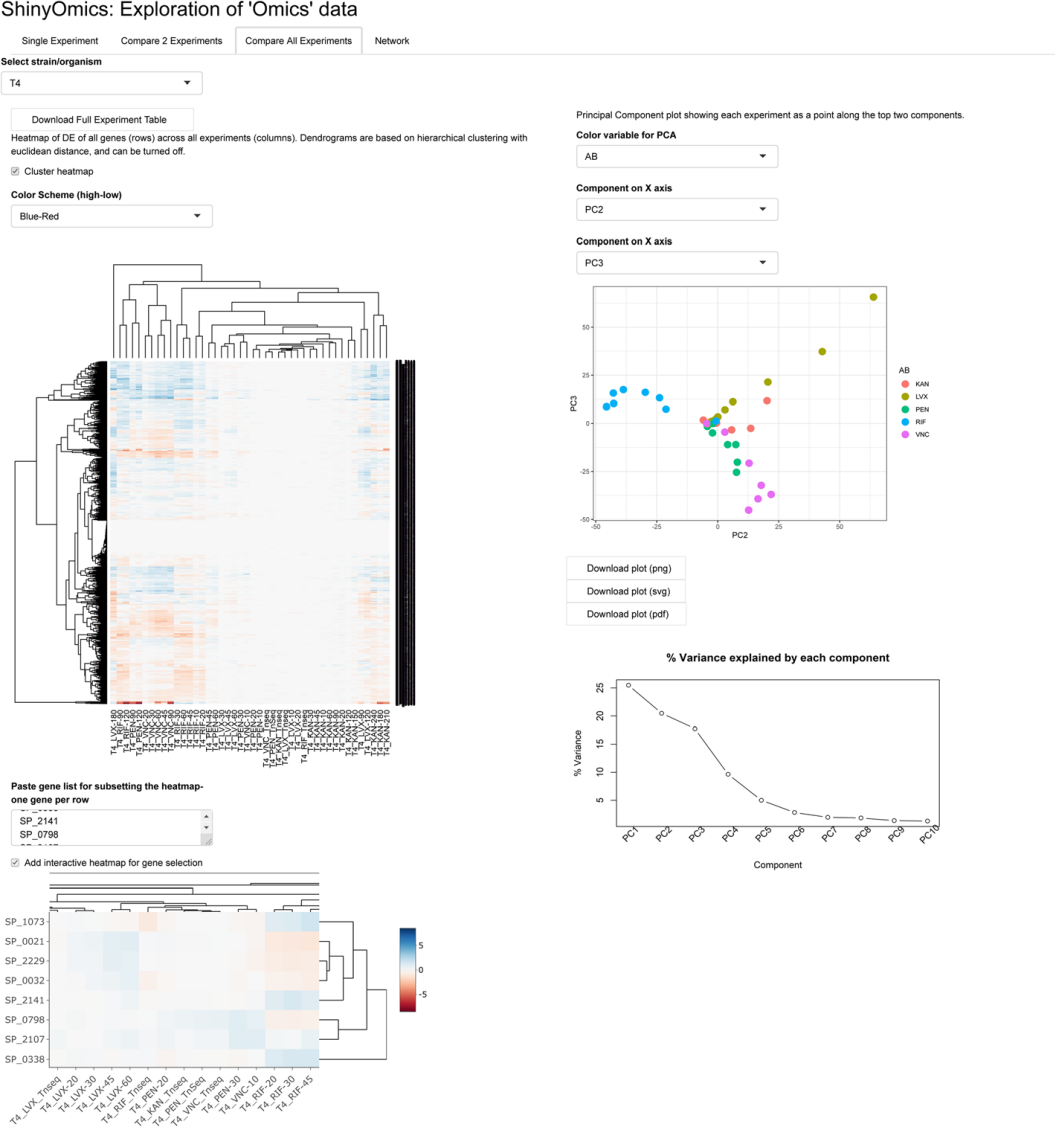
Comparison of all experiments from the same strain. The heatmap shows DE of all experiments included in the experiment sheet for a specific strain (T4: TIGR4). The dendrogram on the heatmap and the PCA (colored by antibiotic) shows that the RNA synthesis inhibitor rifampicin (RIF) is most dissimilar to other antibiotics. AB: antibiotic. KAN: Kanamycin. LVX: Levofloxacin. VNC: Vancomycin. PEN: Penicillin

To identify general patterns across many experimental conditions, the Compare All Experiments panel can be used (Fig. 3). On the left of this panel, a heatmap shows all genes across all conditions, with optional dendrograms showing hierarchical clustering. The heatmap on the bottom is interactive, and shows only a user-specified set of genes, and conditions. On the right side of the panel, principal component analysis (PCA) results are visualized. The first scatter plot shows all experiments on any combination of the top 10 principal components. The user can select which components to plot, and a metadata variable to color the points by (e.g. in order to see whether the experiments are separated by antibiotic, one can select “AB” as the color variable in the pre-loaded dataset). For instance, Fig. 3 shows clear separation of Rifampicin from the other 4 antibiotics. Rifampicin, being an RNA synthesis inhibitor, elicits the most dramatic changes in expression out of the 5 antibiotics included. The last plot shows the percent variance explained by each principal component. The informative components will be those that explain more of the variance in the data. A common way of selecting important components is to look for an ‘elbow’ in the last plot (i.e. a relatively clear point on a line where the slope changes drastically) and consider the components before the elbow [41].

**Fig. 4:**
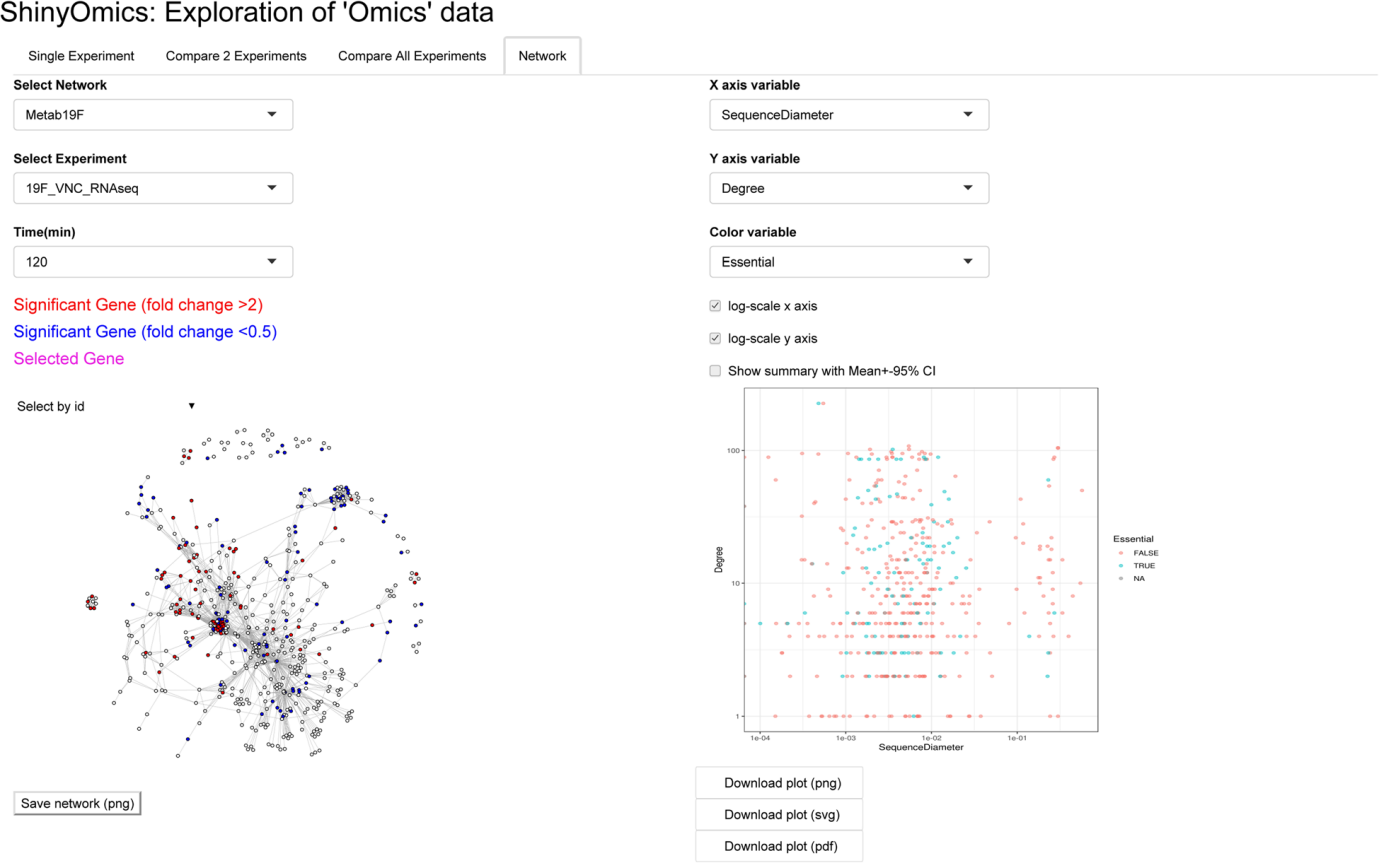
Network visualization of significant DE. The selectors on the upper left allow the user to select a network to display, and a specific experiment and timepoint to overlay. Each gene is a node, and links are defined by the type of network used. The 19F Metabolic (“Metab19F”) network has two genes linked, if their gene products participate in the same reaction, or subsequent reactions in the metabolism of 19F. In the Vancomycin experiment shown (at 120 min), significantly up- and down-regulated genes appear as red and blue nodes respectively. The selectors on the right help generate a scatter plot (lower right) that can relate network-related information (e.g. network degree) to metadata. In the example plot, degree is plotted against sequence diameter i.e. variability of homologous sequences across different strains of *S. pneumoniae*

In order to evaluate whether genes with for instance significant DE (DEGs) or dW are related to one another in a network context, the last panel (Network) allows visualization of a user supplied network of genes. Common types of biologically meaningful networks include protein-protein interaction [42], transcription regulatory [43] metabolic [44] and genetic interaction [45] networks. Depending on the organism, these networks can be manually curated, inferred bioinformatically [46–48], or might already be experimentally mapped out. The preloaded metabolic networks were generated by Jensen et al. [18]. It is also important to keep in mind what kind of network is being used, in order to draw meaningful conclusions from the network analysis. For example, all DEGs localizing on a certain part of the transcription regulatory network may be a result of the DEGs belonging to the same regulon. However, the same phenomenon on a metabolic network may mean a specific metabolic pathway is being activated, which would imply a functional relationship between DEGs. The panel allows the user to select the experiment, timepoint and network, leading to DEGs marked on the network as red and blue nodes for up- and down-regulation respectively. On the example metabolic network of *S. pneumoniae* 19F (initially generated in [18]), the 120-min VNC response is overlaid (Fig. 4). It is possible to pick out numerous groups of interconnected genes that are up- or down-regulated together, although there are also examples of upregulated genes being adjacent to downregulated or non-DE genes. On the left, the network itself will be visualized in an interactive plot that allows zooming, selecting and dragging of nodes. On the right, a set of selectors allow for a custom scatter plot to be made, relating network characteristics of nodes (e.g. degree) to DE or any other metadata supplied by the user. As an example, network degree is plotted against sequence diameter (how variable the sequence is across multiple strains of *S. pneumoniae*), and genes are colored by whether or not they are essential in 19F (Fig. 4), showing a lack of relationship between these variables. Similar to scatter plots in the other panels, this plot is also brushable, and brushed points are displayed in the table below.

## Conclusions

While genome-wide profiling can be incredibly valuable in a variety of applications, initial exploratory analysis of large datasets can be a daunting task. For instance, enumerating the DE of each gene with tools such as DESeq2 is a necessary but insufficient step in such analyses. ShinyOmics is a simple platform for facilitating initial exploratory analysis of omic-profiling data and hypothesis generating. The emphasis on relating genome-wide profiling to custom, user supplied metadata enables the user to make functional associations between any set of features of genes. Moreover, ShinyOmics serves as a convenient data management and sharing tool. Deploying an instance of ShinyOmics with data from a new study results in an interactive supplement for research articles or presentations. For example, a modified version of ShinyOmics accompanying a manuscript with the full antibiotic response dataset from [33] can be found at [49].

## Availability and requirements

Project name: ShinyOmics

Project home page: https://github.com/dsurujon/ShinyOmics

Operating system: Platform independent

Programming language: R (v.3.4.3)

Other requirements: ggplot2 v.3.2.0, visNetwork v.2.0.7, RColorBrewer v.1.1, igraph v.1.2.4, heatmaply v.0.16.0, shinyHeatmaply v.0.1.0, shiny v.1.3.2

License: Affero GPLv3

Any restrictions to use by non-academics: None

## Supplementary information

**Additional file 1: Table S1.** Metadata variables included in the example application, and their descriptions. **Figure S1.** Lack of overlap between different omics data. A. For the TIGR4 KAN experiment, RNA-Seq (Experiment 1) is plotted against Tn-seq (Experiment 2). B. For the M. tuberculosis hypoxia experiment, microarray data (Experiment1) is plotted against proteomics data (Experiment 2).

## Data Availability

The example dataset, user guide and a code for ShinyOmics can be found in the github ShinyOmics repository, https://github.com/dsurujon/ShinyOmics, or as a capsule on CodeOcean [50] An example of the application can be accessed at the URL http://bioinformatics.bc.edu/shiny/ShinyOmics/.

## Abbreviations

DE: Differential expression
DEG: Differentially expressed gene
dW: Difference in fitness
KAN: Kanamycin
LVX: Levofloxacin
PEN: Penicillin
RIF: Rifampicin
VNC: Vancomycin
