# Supplementary material for: ShinyOmics: collaborative exploration of omics-data
Source: BMC Bioinformatics. 2020 Jan 17;21:22. doi: 10.1186/s12859-020-3360-x (PMC6969480; doi:10.1186/s12859-020-3360-x)

**Supplementary Material for ShinyOmics: Collaborative Exploration of Omics Data**

Table S1. Metadata variables included in the example application, and their descriptions

| **Organism** | **Metadata Variable** | **Description** |
| --- | --- | --- |
| *S. pneumonaie* T4/19F | Tag1 | Primary functional tag |
|  | Tag2 | Secondary functional tag (if present) |
|  | Tag3 | Tertiary functional tag (if present) |
|  | Category1 | Primary functional category |
|  | Category2 | Secondary functional category |
|  | Category3 | Tertiary functional category |
|  | No..of.Categories | Number of functional tags/categories a gene belongs to (maximum 3) |
|  | SequenceDiameter | Average pairwise distance between homologs of the same gene across different strains |
|  | SequencePrevalence | Number of strains that have a homolog of the gene |
|  | Essential | Whether the gene is essential |
|  | ExpressionPlasticity | Variability in expression across different experimental conditions |
|  | Location.Tag | Subcellular compartment the gene product localizes to |
|  | Gene.Name | Common name of the gene or gene product |
|  | Gene.Description | Description of the gene or gene product |
| *M. tuberculosis* | start | Gene start position on the chromosome |
|  | end | Gene end position on the chromosome |
|  | strand | Gene strand |
|  | na_length | Length of gene (nucleotide) |
|  | gene.name | Common name of the gene or gene product |
|  | product | Description of the gene or gene product |

Figure S1. Lack of overlap between different omics data. A. For the TIGR4 KAN experiment, RNA-Seq (Experiment 1) is plotted against Tn-seq (Experiment 2). B. For the M. tuberculosis hypoxia experiment, microarray data (Experiment1) is plotted against proteomics data (Experiment 2).


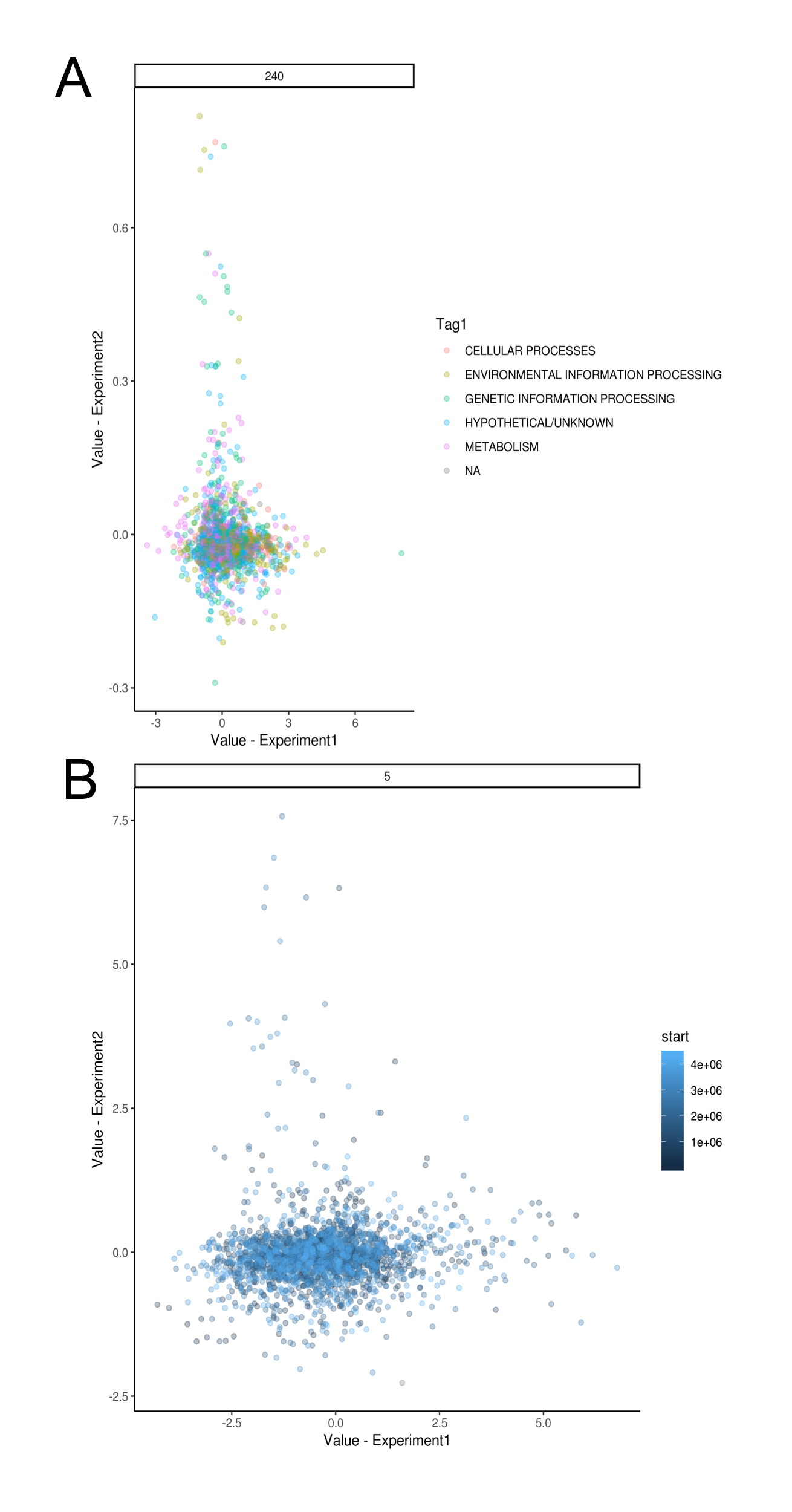

Supplement: Supplementary file 1 — Additional file 1: Table S1. Metadata variables included in the example application, and their descriptions. Figure S1. Lack of overlap between different omics data. A. For the TIGR4 KAN experiment, RNA-Seq (Experiment 1) is plotted against Tn-seq (Experiment 2). B. For the M. tuberculosis hypoxia experiment, microarray data (Experiment1) is plotted against proteomics data (Experiment 2). [file 12859_2020_3360_MOESM1_ESM.docx]
